# Phantoms for Quantitative Body MRI: a review and discussion of the phantom value

**DOI:** 10.1007/s10334-024-01181-8

**Published:** 2024-06-19

**Authors:** Kathryn E. Keenan, Kalina V. Jordanova, Stephen E. Ogier, Daiki Tamada, Natalie Bruhwiler, Jitka Starekova, Jon Riek, Paul J. McCracken, Diego Hernando

**Affiliations:** 1 Physical Measurement Laboratory, National Institute of Standards and Technology, NIST, 325 Broadway, Boulder, CO 80305 USA; 2 Department of Physics, University of Colorado Boulder, Boulder, CO USA; 3 University of Wisconsin, Madison, WI USA; 4 ICON Plc, Blue Bell, PA USA

**Keywords:** Magnetic Resonance Imaging, Phantoms, Clinical Trial

## Abstract

In this paper, we review the value of phantoms for body MRI in the context of their uses for quantitative MRI methods research, clinical trials, and clinical imaging. Certain uses of phantoms are common throughout the body MRI community, including measuring bias, assessing reproducibility, and training. In addition to these uses, phantoms in body MRI methods research are used for novel methods development and the design of motion compensation and mitigation techniques. For clinical trials, phantoms are an essential part of quality management strategies, facilitating the conduct of ethically sound, reliable, and regulatorily compliant clinical research of both novel MRI methods and therapeutic agents. In the clinic, phantoms are used for development of protocols, mitigation of cost, quality control, and radiotherapy. We briefly review phantoms developed for quantitative body MRI, and finally, we review open questions regarding the most effective use of a phantom for body MRI.

## Introduction

Body MRI includes multiple quantitative methods at various stages of development, validation, and dissemination. Quantitative MRI methods measure properties intrinsic to tissue on a per-voxel basis. Relevant quantitative body MRI methods and metrics focus on stiffness [1], proton density fat fraction (PDFF) [2], diffusion (ADC) [3], perfusion [4], susceptibility [5], dynamic imaging [6], and relaxometry (e.g., T1, T2, T2*, R2, R2*) [7]. For development of these methods, it is necessary to demonstrate low bias and high reproducibility across systems. Once a method is implemented in a clinical trial or clinical practice, it is necessary to demonstrate continued reproducibility across multiple sites, over time, and following system changes.

An MRI phantom, also known as a reference object, serves as a versatile tool for evaluating bias and reproducibility in quantitative measurements. MRI research groups use phantoms to validate new MRI quantitative methods and conduct multi-site assessments of bias and reproducibility [8, 9]. Furthermore, phantoms are used in imaging-based clinical trials, encompassing assessments of the clinical value of MRI methods [10, 11] and investigations into therapeutic agents [12]. Beyond MRI methods research, phantoms play a crucial role in quality control (QC) measurements on scanners and serve as educational tools.

For body MRI, phantoms have been developed to evaluate specific quantitative MRI techniques, for example, elastography (MRE), chemical shift-encoded proton density fat fraction mapping [13–15], quantification of diffusion [16–18] and perfusion [19–21], susceptibility mapping [5], dynamic imaging [22], and relaxometry [8, 23, 24]. Given the challenges of body MRI in the presence of physiological motion, many phantoms used by this community simulate motion found within the torso, such as respiratory, cardiac, and blood flow [16, 17, 23, 25–32]. MRI methods research often uses a broad range of phantoms, including custom-developed and home-built phantoms. Additionally, phantoms relevant to body MRI are commercially available from companies such as CaliberMRI, Calimetrix, CIRS (Sun Nuclear), Gold Standard Phantoms, Modus Medical Devices, Resonance Health, Resoundant, and Spectronic Medical.

In this paper, we review the value of phantoms for body MRI in the context of their uses for quantitative MRI methods research, clinical trials, and clinical imaging. Then, we briefly review phantoms developed for quantitative body MRI across applications and discuss their availability. Finally, we review open questions regarding the most effective use of a phantom.

## What is the value of a phantom?

**Fig. 1:**
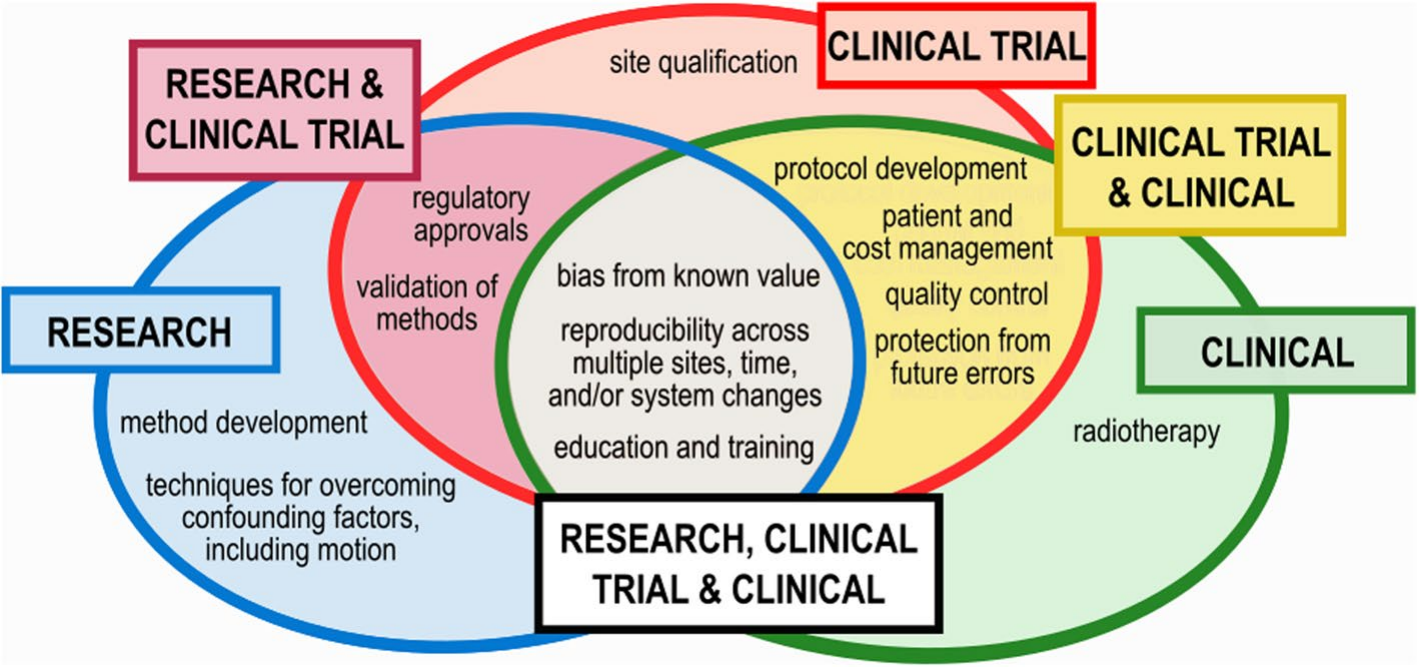
A summary of the key uses of phantoms identified in this paper, across three categories: MRI methods research (“research”), clinical trials, and clinical use. Several phantom uses overlap across the categories; for example, phantoms are used in clinical trials and in the clinic for protocol development, patient and cost management, quality control, and protection from future errors. In this paper we review these uses: first common uses across all categories, then uses in research and clinical trials or clinical trials and clinical, and finally those uses that are predominately limited to one of MRI methods research, clinical trials, or clinical practice

Value is defined by the usefulness of an object. Considering the value of phantoms for quantitative body MRI, we identified several uses of phantoms across MRI methods research, clinical trials, and clinical use (Fig. 1). Phantoms are used to measure and limit bias in MRI data. Periodic use of phantoms is necessary for maintaining reproducibility of MRI data by ensuring that scanners are operating correctly, and sites have the hardware, software, and technical capability to produce the required imaging. Additionally, phantoms are increasingly used for education and training.

One clear demonstration of value is the use of phantoms in the accreditation necessary for medical insurance reimbursement. In the United States, the American College of Radiology (ACR) accreditation program verifies the quality of MRI services provided by accredited institutions [33] using phantoms designed by the ACR (for phantom details, see Ref. [34]). The annual evaluation includes assessment of high-contrast spatial resolution, distance measurements and accuracy, low-contrast detectability, signal uniformity, image ghosting ratio, slice position accuracy, slice thickness accuracy, and image artifacts [35]. Based on the results, medical physicists and service engineers can identify and correct performance issues, leading to consistent image quality [36]. For example, when used twice-monthly (i.e., beyond the recommended use), the ACR MRI phantom improved service engineer workflow [36]. Throughout the world, there are similar recommendations from radiological societies and equipment vendors to use phantoms for achieving image quality [37, 38].

Accreditation, improvements in image quality, and service engineer workflow illustrate the value of a phantom for MRI system assessment. Body MRI methods measure many quantitative properties of tissue, which benefit further from the use of the corresponding quantitative phantoms. The Quantitative Imaging Biomarkers Alliance (QIBA), established in 2007 by the Radiological Society of North America (RSNA), recommended the development and use of phantoms in quantitative imaging for quality control, multi-center studies, and standardization [39]. Here we review the uses of phantoms: first common uses across all categories, then specifically in MRI methods research, in clinical trials, and finally in clinical practice.

### Common uses of phantoms in quantitative body MRI

Certain uses of phantoms are common throughout the MRI community, including for body MRI. These are measuring bias, assessing reproducibility, and training.

#### Measuring bias and assessing reproducibility

It is important to identify, understand and limit any systematic measurement errors (bias) of a method. Quantitative phantoms are key to measuring bias in quantitative MRI techniques [40]. Given the known value to be measured in the phantoms (known either by construction or based on reference measurements), the measurement bias can be determined.

Reproducibility is concerned with the ability of a method to measure the same feature under changing conditions that could be expected in the clinic (e.g., across multiple sites, over time, or using different scanners) [41]. Importantly, reproducibility is frequently assessed along with the bias to demonstrate a method is suitable for in vivo use [12, 41–43]. Phantoms play an essential role in evaluating the reproducibility of MRI, particularly in quantitative mapping [8]. Phantoms are useful for reproducibility assessment because they can mitigate unwanted confounders in MR measurements that can introduce variability in the MR signals, such as physiological motion, diurnal variation, and prandial changes. Further, the same phantom (or nearly identical replicas) can be easily shipped to multiple sites. Nearly identical replica phantoms can be constructed using the same batch of materials, and the reference values can be obtained by a central service, as in the case of the commercial, spherical system phantom [44], or through measurements of all phantoms by a central site, similar to Schneider et al. [13]. Then, any observed variation in the phantoms can be considered when assessing the reproducibility of quantitative methods. Numerous studies have corroborated the effectiveness of MRI phantoms in reproducibility studies. Phantoms with materials designed for a particular application, such as T1 and T2 measurement, are widely used to evaluate reproducibility [5, 45–49]. MRI phantoms are essential in multi-center studies, as they provide a standardized approach for assessing reproducibility across different platforms, which is vital for improving diagnostic accuracy [14, 22, 50–53]. Furthermore, long-term evaluation of MRI system stability, as enabled by phantoms with appropriate shelf-life properties, is essential for the deployment of quantitative MRI [13].

#### Education and training

MRI phantoms are increasingly used for educational and training purposes [54]. This is driven by several factors, including the need to reduce the use of volunteers, to address time constraints on training, and to ensure uniformity in training. The recent COVID-19 pandemic and associated restrictions have further encouraged the adoption of phantoms for training.

Qualitative phantoms developed for multiple imaging modalities can produce realistic MR images by mimicking organs, vasculature, and common pathologies, including tumors. These are suitable for teaching and training, including for MRI-guided interventions [25, 28, 55–58]. Furthermore, the phantom is a convenient device to scan as part of a training exercise when using new tools or techniques, such as MRE equipment.

Quantitative MRI phantoms can be used to facilitate the teaching of MRI principles and MRI scanning to radiologists, technologists, and engineering and medical physics students. They can be used to demonstrate different quantitative MRI techniques and ensure that trainees obtain the expected value, especially when learning new protocols. For example, a simple ice water phantom (with a predictable diffusion coefficient due to the effective temperature control) was used to train sites using different scanners, measurement protocols, and software tools on the correct measurement of apparent diffusion coefficient [59]. Finally, quantitative MRI phantoms can also be used to identify and correct common MRI artifacts and quantitative confounding factors.

### Value of phantoms for quantitative body MRI methods research

In addition to the previously mentioned uses, phantoms are used in body MRI methods research for novel methods development and the design of techniques to overcome confounding factors.

#### Novel methods development

The first phantom use-case often occurs during the design and validation phase of a new method. While many research phantoms are designed to be replicated by other sites and groups [24], the phantoms used for initial methods development can be home-made or single-use as long as they are sufficiently well-characterized. Although this use-case typically consists of a small user base (e.g., a single research group), the downstream effects of the phantom can be quite influential if the method developed using it makes it to the clinic. In such cases, it is possible that the phantom itself will be further developed as the method is disseminated. Examples of phantoms for body MRI that started as single-use research phantoms and have progressed to commercially available products include the PDFF phantoms [14, 43, 60] made by Calimetrix (Madison, WI, USA) and the MRE phantoms [61] made by Resoundant (Rochester, MN, USA).

At this early stage in methods development, phantoms are also used to evaluate repeatability [8]. Repeatability concerns the ability of a method to measure the same feature under nearly identical conditions [41]. Test–retest repeatability in a phantom is important to assess basic stability of a method. If a method has poor repeatability in a phantom, it is not ready for in vivo use. However, to understand the true repeatability of a method we recommend moving beyond a phantom and testing repeatability in vivo.

#### Techniques for overcoming confounding factors

Many quantitative body MRI methods involve measuring multiple quantitative properties of one tissue or measuring a single parameter that is impacted by other confounding factors. For example, multiple MRI parameters are used in the diagnosis and monitoring of diffuse liver disease. Fat, iron, and fibrosis can all affect the MR signal in the liver in different ways, as measured by PDFF, R2 or R2*, and T1, respectively. However, these parameters can confound each other if not adequately addressed. For example, R2* and T1 relaxation can confound PDFF quantification, fat can confound R2* quantification, and both fat and R2* can confound T1 quantification. Mixed quantitative MRI phantoms [60] are a valuable tool for assessing the measurement bias in these situations. By providing a controlled environment in which the effects of different tissue properties can be studied independently and in combination, mixed phantoms contribute to quantitative MRI sequences with less bias for the diagnosis and monitoring of liver diseases. For example, PDFF and R2* are frequently assessed using the same phantoms [14, 46, 50]. In prostate imaging, phantoms facilitate evaluation for multiparametric MRI, such as ADC, T1, R2*, radiomics features, and ROI volumes [47, 62–64]_._ Similarly, in breast imaging, phantoms contain fat to assess fat suppression, materials to modulate ADC and T1, and components to mimic dynamic contrast effects [22, 65, 66].

Motion is another confounding factor in body imaging applications. Numerous phantoms designed to emulate physiological motion have been proposed to validate imaging techniques. Numerical, or digital, phantoms can be used to evaluate the effects of respiratory motion for a variety of imaging applications and conditions that can be prohibitively difficult to perform in vivo [67]. Phantoms that mimic respiratory motion can be used to evaluate the degradation of image quality caused by bulk motion [27, 68], assess the quantitative accuracy of diffusion measurements [16], and develop new methods to address these issues. As an example, in the studies by Geng et al., the researchers used a compressive motion phantom (3D phantom of liver and pancreas) to simulate the impact of cardiovascular pulsation and breathing motion in diffusion MRI of the abdomen. They found that motion could cause a significant bias in ADC values in some parts of the liver and pancreas. This finding contributed to the development of motion-robust DWI sequences, with the potential to improve diagnostics in these organs [17, 18]. Similarly, Zhong et al. used a home-built motion phantom to assess the performance of PDFF quantification in chemical shift encoded MRI [23]. A motion phantom is not only effective in validating the accuracy of flow measurements gathered through phase-contrast MRI [69], but it also proves useful for validating strain measurement using cine phase-contrast MRI [30]. Although phantoms cannot replicate the complexity of physiological motion observed in vivo, the ability to control (and stop) motion makes phantoms invaluable tools in body MRI methods research.

### Value of phantoms for quantitative body MRI in clinical trials

Clinical trials are used to demonstrate the safety and efficacy of a therapeutic agent or a device, and imaging is one frequently employed tool that supports this determination. Clinical trials are also used to demonstrate the clinical value of new imaging methods. Clinical research organizations (CROs) are often engaged to execute all or part of a clinical trial for pharmaceutical, biotechnology, or medical device companies. In addition to the common use cases for phantoms, the use of quantitative MRI phantoms in clinical trials reduces the risk of scanning a participant incorrectly, erroneously including or excluding a patient from participating in a clinical trial, and reporting values or changes in quantitative imaging measurements that are due to changes in equipment or imaging technique rather than changes due to therapeutic intervention.

Because clinical trials are usually carried out by multiple clinical investigators at different locations around the world, there is a great need to ensure consistency throughout the range of imaging equipment and personnel that will participate in the clinical trial. Quantitative MRI phantoms, coupled with training and centralized image review, are an essential tool in providing this consistency. Before an imaging site begins scanning patients for trial eligibility or baseline measurements, a site qualification process is often followed. Site qualification usually involves obtaining images of the quantitative MRI phantoms and submitting them to the CRO. Then, the CRO will ensure that all the imaging parameters required to calculate the quantitative measurements are correct. To complete site qualification, the submitted images of the phantoms are used to characterize the performance of the scanner by confirming that the quantitative measurements in the phantoms are within expected ranges. During a trial, the phantoms can be used to determine if the performance of the scanner changes over time (longitudinal QC).

#### Development of protocols

As described earlier, phantoms can be used to define and test protocols prior to actual use in the clinic or a clinical trial. Both qualitative and quantitative MRI phantoms are useful for developing or updating body MRI protocols, because they provide a consistent and reproducible environment for testing and optimizing imaging parameters. For example, a recent study used an abdominal phantom to design a standardized workflow and multimodal imaging pipeline to detect liver lesions [70].

As institutional review boards increasingly limit the use of volunteers for site qualification, the use of phantoms for testing and optimizing protocols prior to patient involvement limits the need for subject scans and reduces site liability while ensuring tested and validated protocols [54]. Preliminary testing using phantoms minimizes the reliance on animal and human subjects, thereby reducing ethical dilemmas related to protocol testing and development.

#### Patient and cost management

Within the context of a clinical trial, the results of quantitative MRI may be used to help determine if a patient is eligible to participate in the clinical trial. For example, the eligibility criteria in clinical trials investigating therapeutic agents to treat steatotic liver disease often include a liver PDFF above a certain threshold (typically 6–10%) at the beginning for the trial. Additionally, these same quantitative MRI measurements may also contribute to determining the safety and/or efficacy of the intervention being studied in the clinical trial. In the steatotic liver example, many clinical trials have an efficacy endpoint that is defined as the change over time in hepatic steatosis as measured by PDFF [71].

Phantoms have measurable value when used to ensure the optimal functionality of MRI systems and data integrity of scans. Cost is a significant factor in clinical trials as well as healthcare. Using phantoms to prospectively and preventively identify MRI acquisition errors limits the need to repeat patient imaging at later dates, mitigating associated costs and delays.

#### Facilitation of regulatory approvals

Whether primary, secondary, or exploratory endpoints, comprehensive phantom study data substantiate the claims presented during the regulatory approval process, thereby enhancing the credibility and acceptability of the data. Detailed documentation and results from phantom studies are critical evidence during regulatory submissions and reviews. This is true for both novel MRI methods and therapeutic agents. For example, phantom metrics for the measurement bias and repeatability were cited in the FDA 510(k) filing for Perspectum LiverMultiScan (LMSv3) [72]. Similarly, phantoms supported the FDA 510(k) filings for Siemens' LiverLab [73], Philips’ mDIXON-Quant [74] and GE Healthcare’s IDEAL IQ [75].

As regulatory support for new therapeutics and biomarkers is pursued, the deployment of phantoms allows for standardized benchmarking, enabling the systematic QC of MRI system performance across different studies, sites, timing, and therapeutic agents. The FDA has guidance for optimizing the quality of imaging data from clinical trials to support therapeutic agents [76] and for radiological devices that include quantitative imaging functions [77]. Both documents describe the use of phantoms to support the claims.

#### Case study: MASH clinical trials

In the field of metabolic dysfunction-associated steatohepatitis (MASH), MRI is a preferred clinical trial tool that can non-invasively measure liver fat (e.g., via PDFF) and fibrosis (e.g., via MRE) [78]. Although regulatory agencies require the use of a percutaneous liver biopsy in later stage clinical trials to determine efficacy, imaging may be used for this purpose through Phase IIa trials. In these imaging-based trials, phantoms that cover a wide range of PDFF values are used for site qualification, to assess protocol adherence, and for proactive identification of the need to rescan a patient. These phantoms can be scanned alone (for site qualification) or with a patient/participant during the clinical trial to help determine if the imaging was performed correctly.

**Fig. 2:**
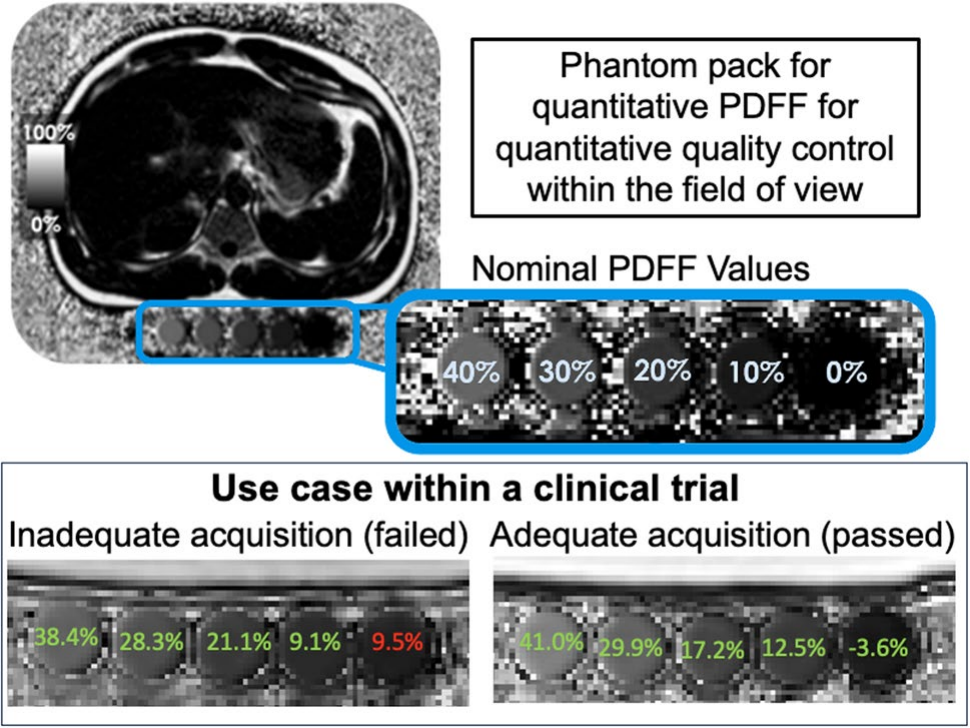
Proton density fat fraction (PDFF) phantom scanned below a patient during a metabolic dysfunction-associated steatohepatitis (MASH) clinical trial. The nominal PDFF values are shown along with an inadequate acquisition followed by an adequate acquisition

Typically, image data collection issues are identified because the PDFF map has incorrect quantitative values in the phantom (Fig. 2). For example, the PDFF values may be out of range, which is identified based on the expected fat fraction values (and ranges) for each of the phantom vials. If during image review (or QC), the phantom values are out of range, the CRO will identify the root cause of the issue and provide feedback to the data collection site. The cause may be incorrectly acquired data due to poor shimming, inaccurate pre-scan, or issues with the phase correction baseline data. Acquisition issues are another possible source of error; these include incorrect flip angle, incorrect echo times, incorrect phase encoding direction, or the use of fat saturation.

Finally, image artifacts in both the acquisition and reconstruction may be identified using the phantom. One common artifact is motion, which can often occur in patients who are unable to hold their breath. It can be easier to identify the artifact in the phantom than in the participant. In the case of a fat/water swap, the swap is obvious in a phantom where there are expected fat content for different locations. Some scanners, under specific conditions, or in persons with low body fat, may swap fat/water in the qualification or patient scans and this needs to be corrected.

The use of phantoms to identify protocol errors and image artifacts enables conversations with imaging sites on the correctness of the protocol and data collection.

### Value of phantoms for clinical quantitative body MRI

Both qualitative and quantitative phantoms are used in the clinic for the purposes of quantitative body MRI. In addition to the common uses, phantoms are used for development of protocols and mitigation of cost, which are detailed in the previous section on use for clinical trials. Here, we describe the use of phantoms in the clinic for quality control and radiotherapy.

#### Quality control

MRI phantoms are widely used for quality control in the clinic, which includes testing newly installed or revised MRI software or hardware and routine testing. Importantly, phantoms provide a consistent and reproducible environment for these tests, for optimizing imaging parameters, and for assessing newly installed sequences or equipment [37, 38]. Further, accreditation agencies such as the ACR in the United States mandate MRI phantom-based QC as part of their accreditation process of MRI procedures.

Several vendors include quantitative and qualitative phantoms as part of their body MRI product packages. For example, Resonance Health's FerriScan phantom is a specially designed quantitative phantom used to ensure the standardization and accuracy of the FerriScan R2 mapping protocol setup [79]. This phantom is essential for ensuring that FerriScan, developed for quantifying liver iron in MRI, provides minimally biased R2 values for quantifying liver iron concentration. Another example, a mixed-function MRE phantom provided by Resoundant, is used to verify that Resoundant’s MRE system is installed and functioning properly [61]. This includes qualitative evaluation, such as verifying the generation of shear waves, wave images, and elastograms, as well as longitudinal assessment of measurement reproducibility.

#### Radiotherapy treatment design and planning

Recently, MRI has been widely adopted in radiotherapy for treatment planning, primarily due to its superior image contrast, and phantoms are essential tools in this setting. Image distortions could introduce bias in tracking anatomies of interest. Lattice phantoms, which possess a regular and well-defined structure, have been developed for this purpose. These phantoms can assess image distortions caused by B_0_ inhomogeneity, gradient nonlinearity, and eddy currents that could impact radiotherapy planning [80–87]. Additionally, motion phantoms are increasingly used in the field of body radiotherapy. Similar to lattice phantoms, motion phantoms play a crucial role in evaluating the motion tracking characteristics of systems [25, 29, 31]. The performance of an MR-Linac system when treating moving targets was evaluated using a dynamic motion phantom, and results showed that the measured dosage accuracy was clinically acceptable [88]. Motion phantoms were also used to evaluate MRI of the liver on an MR-Linac [89].

Quantitative MRI methods can be paired with radiotherapy treatment to assess response to treatment and progression of disease [90]. As a result, phantoms have been used to measure the reproducibility of ADC on MR-Linac [91, 92] and MRI systems used by radiotherapy teams [48]. Given the necessity of phantoms for ensuring proper radiation dose, the MR-Linac community has readily adopted the use of phantoms for MRI measurements.

## Phantoms for quantitative body MRI

### Available phantoms

A selection of body phantoms that are either commercially available or have published designs can be seen in Table 1. These phantoms are designed for a wide range of applications across body MRI, and Table 1 is organized according to use. The phantoms have a variety of forms, both anthropomorphic and geometric, to be used alone or imaged with a patient. Phantoms that can be imaged with the participant add information as to whether the images were acquired correctly. Some of the included phantoms even accommodate simulated biopsies or subject motion. The selection of phantoms was assembled from the knowledge of the authors, as well as results from PubMed and Google Scholar searches for terms such as “Abdominal MRI Phantom”, “Body MRI Phantom”, “Prostate MRI Phantom”, “Liver MRI Phantom”, and “Geometric Accuracy Phantom MRI”.

**Table 1:** A selection of body MRI phantoms that are either commercially available or have published designs

Category	Description	Designer	Form	Availability	Designed for	Citations
Image Quality	Cylindrical, Image Quality Phantom [34]		Cylindrical	Commercial	Geometric distortion, resolution, slice thickness, image quality	5 +
Image Quality	Cylindrical Geometric Distortion Phantom [97]		Cylindrical	Commercial	Geometric distortion, MRI-guided radiation therapy	5 +
Image Quality	Rectangular Geometric Distortion Phantom [97]		Rectangular	Commercial	Geometric distortion, MRI-guided radiation therapy	5 +
Image Quality	Geometric Distortion Torso Phantom [98]		Torso	Commercial	Geometric distortion, MRI-guided radiation therapy	5 +
Image Quality	3D-Printed Grid Head Phantom [82]	Nousiainen and Mäkelä	Cylindrical	Published Description	Geometric distortion	1–2
Image Quality	Planar Head Distortion Phantom [83]	Orth et al	Lattice	Published Description	Geometric distortion, MRI-guided surgery	0
Image Quality	3D-Printed Grid Torso Phantom [84]	Ramachandran et al	Torso	Published Description	Geometric distortion, MRI-guided radiation therapy	0
Image Quality	Modular Distortion Phantom [85]	Slagowski et al	Modular Blocks	Published Description	Geometric distortion, MRI-guided radiation therapy	0
Image Quality	Rectangular Grid Distortion Phantom [86]	Wang et al	Rectangular	Published Description	Geometric distortion	1–2
Image Quality	Rectangular Rod Distortion Phantom [87]	Yu et al	Rectangular	Published Description	Geometric distortion, MRI-guided surgery	1–2
Interventional	Abdominal Phantom for Biopsy Practice [99]		Anthropomorphic, Biopsy	Commercial	MRI-guided biopsy	1–2
Interventional	Abdominal MRI, CT, and US Phantom for Biopsy Practice [100]		Anthropomorphic, Biopsy	Commercial	MRI-guided biopsy, Image fusion	3–4
Interventional	Phantom for Radiation Therapy QA and Motion Tests [95]		Anthropomorphic, Motion	Commercial	MRI-guided radiation therapy, motion compensation	5 +
Interventional	Phantom for Radiation Therapy Motion Tests [96]		Anthropomorphic, Motion	Commercial	MRI-guided radiation therapy	5 +
Interventional	MR-guided Focused Ultrasound Tumour Phantom [58]	Antoniou et al	Rectangular	Published Description	MRI-guided focused ultrasound	0
Interventional	Interventional MRI, CT, and PET-CT Abdominal Phantom [70]	Bauer et al	Anthropomorphic	Published Description	MRI-guided biopsy	0
Interventional	Liver Thermal Ablation Phantom [101]	Bazrafshan et al	Optical absorption, Liver	Published Description	MRI-guided thermal ablation	0
Interventional	Low-cost Motion Phantom [31]	Chang et al	Rectangular, Motion	Published Description	MRI-guided radiotherapy, motion	0
Interventional	Liver Respiratory Motion Phantom [29]	Geelhand de Merxem et al	Anthropomorphic Liver, Motion	Published Description	MRI-guided radiation therapy	0
Interventional	Interventional Phantom for MRI/CT Fusion [57]	Johnston et al	Rectangular	Published Design	MRI-guided biopsy	0
Interventional	Respiratory Liver Motion Phantom [32]	Maier-Hein et al	Anthropomorphic, Liver, Motion	Published Description	MRI-guided procedures	1–2
Interventional	Flow Liver Phantom [28]	Rethy et al	Anthropomorphic, Liver, Flow	Published Description	MRI planning of laparoscopic ultrasound	0
Interventional	Anthropomorphic Pelvic Phantom [102]	Singhrao et al	Anthropomorphic, Prostate	Published Description	MRI-guided radiation therapy	0
Interventional	Geometric Pelvic Phantom [81]	Sun et al	Anthropomorphic, Prostate	Published Description	MRI-guided radiation therapy, distortion analysis	0
Interventional	Dynamic Phantom with Heart, Lung, and Blood Motion for Initial Validation of MRI Techniques [26]	Swailes et al	Anthropomorphic, Motion	Published Description	Motion compensation, MR-guided procedures	0
Interventional	Pelvic Distortion Phantom [80]	Tanner et al	Rectangular	Published Description	MRI-guided radiation therapy, distortion analysis	1–2
Interventional	Abdominal Motion Phantom for MRI-guided Radiation Therapy [25]	Weidner et al	Anthropomorphic, Motion	Published Description	MRI-guided radiation therapy	0
qMRI	Phantom for Quantitative MRI of the Prostate [103]		Anthropomorphic, Prostate	Commercial	T_1_, T_2_, diffusion	1–2
qMRI	Phantom for Quantitative, Isotropic Diffusion [94]		Spherical	Commercial	Diffusion	5 +
qMRI	Phantoms for Quantitative PDFF, T_1_, T_2_, R2* MRI [60]		Spherical Housing-based (Stand-alone Imaging), Set of Cylinders (in-the-FOV Imaging under the Patient)	Commercial	PDFF, T_1_, T_2_, R2*	5 +
qMRI	Phantom for Shear Wave Live Fibrosis [104]		Cylindrical	Commercial	MRE	1–2
qMRI	Phantom for Quantitative Breast MRI [66]	Keenan et al	Cylinders, Spheres	Commercial	T_1_, T_2_, diffusion, geometric distortion	5 +
qMRI	Spherical General Purpose Phantom [44]	Stupic et al	Spherical	Commercial	T_1_, T_2_, proton density, geometric distortion	5 +
qMRI	Phantom for R2* Quantification [79]		Vials	Commercial	R2*, Liver iron concentration	1–2
qMRI	Phantom for MR Elastography [61]		Cylindrical	Commercial	MRE	1–2
qMRI	Dynamic Hepatocellular Carcinoma Liver Phantom [55]	Ahmad et al	Anthropomorphic, Liver	Published Description	Dynamic contrast enhancement	0
qMRI	Breast qMRI and Flow Phantom [22]	Basukala et al	Cylinders, Spheres	Published Description	T_1_, T_2_, diffusion, flow	0
qMRI	Anthropomorphic Breast Phantom [65]	Freed et al	Anthropomorphic	Published Description	T_1_, T_2_	1–2
qMRI	3D-printed Pancreas Motion Phantom [17]	Geng et al	Anthropomorphic, Motion	Published Description	Diffusion, motion	0
qMRI	3D-printed Pulsatile Flow Liver Phantom [18]	Geng et al	Anthropomorphic, Liver, Flow	Published Description	Diffusion, blood-suppression	0
qMRI	Fat–Water-SPIO Fat Fraction Phantom [93]	Hines et al	Vials	Published Description	PDFF	3–4
qMRI	Fat–Water Phantom [14]	Hernando et al	Vials	Published Description	PDFF	1–2
qMRI	Disposable Point‐of‐care Portable Perfusion Phantom (DP4) [20]	Holland et al	Rectangular	Published Design	Perfusion, quantitative dynamic contrast enhancement MRI	0
qMRI	Linear Motion Phantom [30]	Jensen et al	Rectangular, Motion	Published Description	Cine phase contrast, strain	0
qMRI	Point-of-care Portable Perfusion Phantom (P4) [19]	Kim et al	Rectangular	Published Design	Perfusion, quantitative dynamic contrast Enhancement MRI	1–2
qMRI	DCE Prostate Phantom [105]	Knight et al	Anthropomorphic, Prostate	Published Description	Dynamic contrast-enhanced MRI	1–2
qMRI	Linear Respiratory Motion Diffusion Phantom [16]	Kwee et al	Motion	Published Description	Diffusion, motion	0
qMRI	Collagen-Water-Fat-Gd R2* and Susceptibility Phantom [5]	Li et al	Rectangular	Published Description,	R_2_*, susceptibility	0
qMRI	PDFF-CT Phantom [15]	Pickhardt et al	Cylindrical	Published Description	PDFF	0
qMRI	Radiomics Phantom [53]	Rai et al	Spherical	Published Design	Radiomics	0
qMRI	3D-printed ASL Perfusion Phantom [21]	Wang et al	Rectangular	Published Description	Perfusion	1–2
qMRI	Abdominal Kidney QA Phantom [24]	Wolf et al	Anthropomorphic	Published Design	Conductivity, permittivity, T_1_, and T_2_	1–2
qMRI	Hydrostatic Linear Motion Phantom [23]	Zhong et al	Vials, Motion	Published Description	R2*, motion	0

In the “Description” column, the citation given is the first published description of the phantom or a representative use case if no published description is available. The “Citations” column reports approximate number of uses of the phantom found in the literature

Within a section of Table 1, the phantoms are organized by availability. In the “Availability” column, *Commercial* indicates that the phantom is available for sale. *Published design* indicates that a design with sufficient detail for the reader to build the phantom has been published in a peer-reviewed publication. *Published description* indicates that a design has been described in a peer-reviewed publication, but detailed information to build was not included. In the “Name” column, the citation given is the first published description of the phantom or a representative use case if no published description is available. The “Citations” column reports the approximate number of uses of the phantom found in a literature search from PubMed and Google Scholar, or, for some commercially available phantoms, the manufacturer website provided a list of citations.

**Fig. 3:**
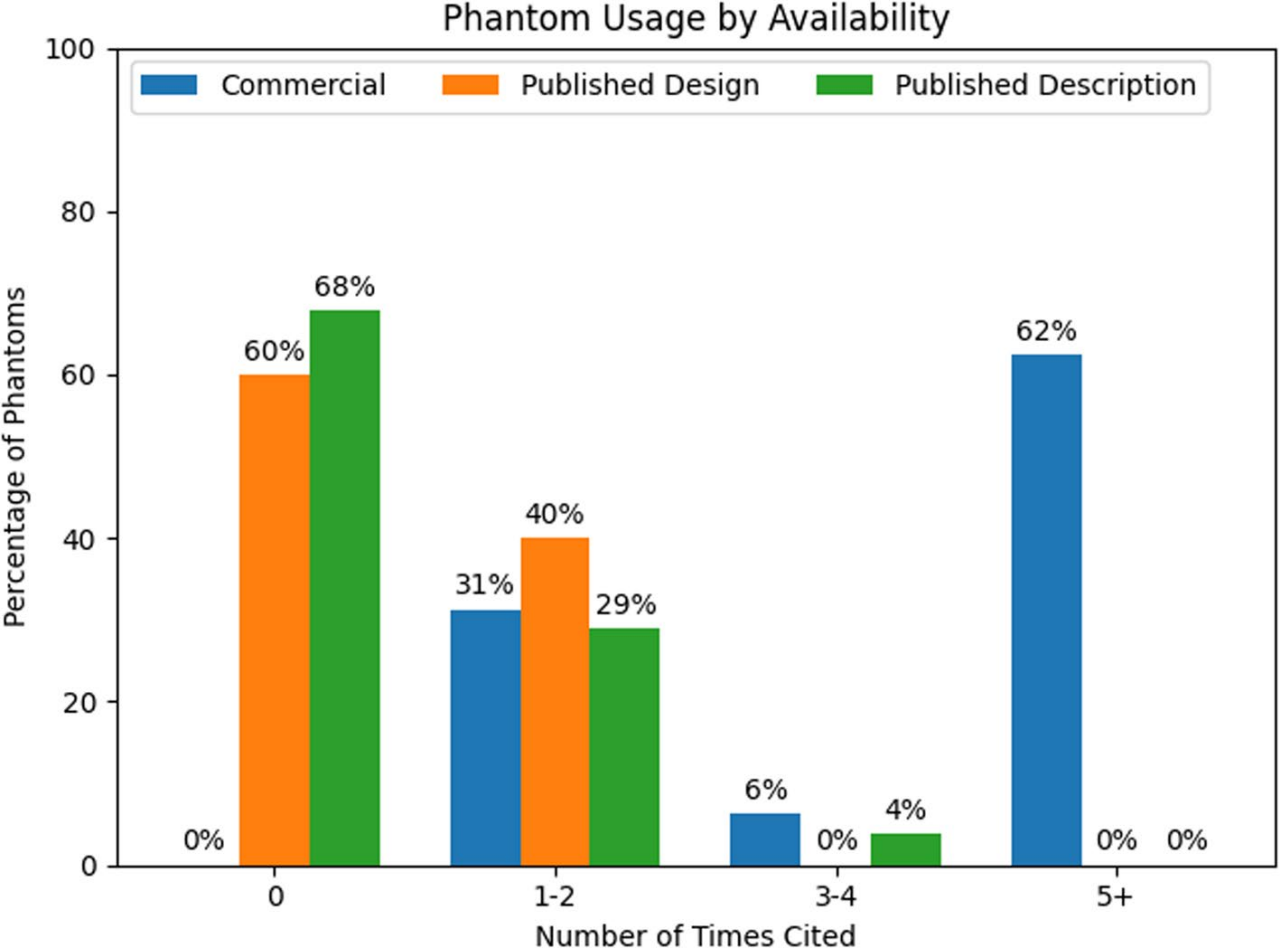
Frequency of phantom usage by phantom availability, as a percentage of the total phantoms in a category. Commercially available phantoms are the most widely used, and phantoms with published designs are more likely to be used than those with only descriptions

Published descriptions have been replicated (e.g., Schneider et al. [13] used the description from Hines et al. [93]); however, commercially available phantoms have more literature citations than the published designs and published descriptions (Fig. 3). Commercially available phantoms with five or more citations include a spherical general purpose phantom for T1, T2, proton density and geometric distortion [44]; a quantitative breast phantom for T1, T2, diffusion and geometric distortion [66]; phantoms for quantitative PDFF, T1, T2, and R2* [14]; phantom for isotropic diffusion [94]; phantoms for radiation therapy motion tests [95, 96]; and a cylindrical image quality phantom for geometric distortion [34].

### Limitations of current phantoms

The currently available phantoms have demonstrated value, as discussed throughout this paper. However, there are limitations of current phantoms that aim to mimic tissue properties, and it is important to understand both the capabilities and limitations of a given phantom.

Phantoms cannot replicate all the MRI-measurable properties of a tissue. For example, it is possible to make a phantom with a physiologically relevant range of T1 and T2 values, including the T1/T2 ratio. Thus far, though, it has not been possible to also represent the ADC in the same T1/T2 phantom. Another example comes from phantoms that mimic liver iron content. It is possible to make R2 phantoms or R2* phantoms. Although phantoms may have a relationship between R2 and R2* that mimics the iron-loaded liver, quantitatively this relationship is generally not the same as observed in vivo.

Quantitative properties and phantom materials can be temperature and field dependent, which can complicate the assessment of accuracy and reproducibility. Diffusion is a temperature dependent parameter, and it is necessary to measure temperature of the phantom during the imaging session. Thermometers can be included in phantoms for this purpose [106]. Relaxation properties of materials that are frequently used in phantoms, such as CuSO4, NiCl2, and MnCl2, vary with field and temperature, and not always in a straightforward manner [44]. As a result, it is important to characterize phantom materials across these conditions.

Finally, tissue relaxation properties are field dependent; the T1 and T2 or R2 and R2* values change with field strength, even between 1.5 and 3 T. Thus far, materials used in phantoms do not have the same field-dependent properties as tissue. For example, commonly used R2* phantoms are based on superparamagnetic iron oxides, where the magnetization is essentially saturated at 1.5 T, and therefore the R2* values are nearly the same between 1.5 and 3 T. In contrast, in vivo R2*, in the presence of liver iron overload, nearly doubles between 1.5 and 3 T.

## Open questions about the use of phantoms

There are many open questions regarding the use of phantoms, in particular for routine use in the clinic. It is known that quantitative measurements change with software versions, pulse sequence changes and hardware changes [107–109]. However, the definition of “routine” in this case is unknown. We do not know when the phantom should be used or at what frequency the phantom measurements are necessary. It is surmised that the stability of a measurement should be tracked over time and deviations treated as a warning to look for root cause errors in the measurement specifications or changes in the measurement system. However, there is minimal literature on which to base recommendations.

For other imaging modalities, the use of phantoms is necessary and clear. Frequently there are recommended or required QC protocols that are used. For example, in any radiation-based method, a phantom is necessary to ensure accuracy of dose. In dual-energy X-ray absorptiometry (DXA), quantitative phantoms are used for daily calibration to ensure that measured bone density (BMD) remains consistent over time [110]. Changes in these measured densities can be used to adjust patient bone densities to minimize the effect of longitudinal scanner variability. Within a multi-center clinical trial, it is also useful to reduce the variability of measurements between sites. This can be accomplished using a cross-site calibration phantom such as the Bona Fide Phantom [110]. By scanning the cross-site calibration phantom at each DXA imaging site, the measured values can be used to adjust the BMD of each participant to substantially reduce the variability observed between different DXA scanners. Cross-site calibration phantoms have also been used as part of an equipment replacement program to minimize equipment downtime [111].

The clinical trial and clinical imaging communities would benefit from clear directions on when to use quantitative MRI phantoms. Such directions would necessarily balance the time required and costs of QC with the benefit derived from making these measurements. Data are needed on the frequency and severity of errors in imaging studies and use of phantoms to identify or possibly correct these errors. Additionally, documentation of limited system downtime due to the use of MRI phantoms is needed. We call on the community to consider these questions when designing studies and publishing findings on the use of phantoms.

## Conclusion

Phantoms for quantitative body MRI have been developed for many purposes and to address the multitude of needs of this community. These phantoms have clear and demonstrated value in MRI methods research, for clinical trials, and in the clinic. For example, phantoms are indispensable for limiting bias and validating the reproducibility of imaging data, which is necessary for validation of new imaging methods, for affirming the validity of findings in clinical trials, and for supporting data-driven decisions in the clinic. Additionally, phantoms are an invaluable tool for education and training of new methods, equipment, and protocols to reduce variances induced by operator error.

Phantoms are an essential part of quality management strategies, facilitating the conduct of ethically sound, reliable, and regulatorily compliant clinical research of both novel MRI methods and therapeutic agents. Sharing data and findings from phantom studies provides transparent communication with researchers, illustrating adherence to quality and consistency. This is particularly important when demonstrating a method is ready for translation to the clinic and necessary to secure regulatory clearance for commercial quantitative MRI. The strategic incorporation of phantoms in body MRI-focused clinical trials not only assures the qualitative and regulatory robustness of the trial but also supports the validity and reliability of the data, which is critical for decisions evaluating the safety and efficacy of investigational therapies.

To support further adoption of quantitative body MRI methods, we recommend that the community study and develop best practices for routine clinical use of phantoms.

## Disclosures

Certain commercial equipment, instruments, or materials are identified in this paper in order to specify the experimental procedure adequately. Such identification is not intended to imply recommendation or endorsement by NIST, nor is it intended to imply that the materials or equipment identified are necessarily the best available for the purpose.

D.H. is co-founder of Calimetrix.

## Data Availability

Data availability statement is not applicable.
